# Cardiovascular Risk in Non-Alcoholic Fatty Liver Disease: Mechanisms and Therapeutic Implications

**DOI:** 10.3390/ijerph16173104

**Published:** 2019-08-26

**Authors:** Claudio Tana, Stefano Ballestri, Fabrizio Ricci, Angelo Di Vincenzo, Andrea Ticinesi, Sabina Gallina, Maria Adele Giamberardino, Francesco Cipollone, Richard Sutton, Roberto Vettor, Artur Fedorowski, Tiziana Meschi

**Affiliations:** 1 Internal Medicine and Critical Subacute Care Unit, Medicine Geriatric-Rehabilitation Department and Department of Medicine and Surgery, University-Hospital of Parma, 43121 Parma, Italy; 2 Internal Medicine Unit, Pavullo Hospital, Azienda USL, 41026 Modena, Italy; 3 Institute for Advanced Biomedical Technologies, Department of Neuroscience, Imaging and Clinical Sciences, University “G. d’Annunzio”, 66100 Chieti, Italy; 4 Department of Clinical Sciences, Faculty of Medicine, Lund University, SE-205 02 Malmö, Sweden; 5 Fondazione Villa Serena per la Ricerca, Viale Leonardo Petruzzi, 42-65013 Città Sant’Angelo, Italy; 6 Geriatrics Clinic, Department of Medicine and Science of Aging and Ce.S.I.-MeT, “G. d’Annunzio” University of Chieti, 66100 Chieti, Italy; 7 European Center of Excellence on Hypertension, Dyslipidemia and Atherosclerosis and Department of Medicine and Science of Aging, “G. d’Annunzio” University, 66100 Chieti, Italy; 8 National Heart and Lung Institute, Imperial College, Hammersmith Hospital Campus, Ducane Road, London W12 0NN, UK; 9 Center for the Study and the Integrated Treatment of Obesity–Department of Medicine—University of Padova, 35122 Padua, Italy; 10 Clinical Research Center, Dept, of Cardiology, Skåne University Hospital, SE-205 02 Malmö, Sweden

**Keywords:** non-alcoholic fatty liver disease, cardiovascular disease, metabolic syndrome, cardiovascular risk factors

## Abstract

New evidence suggests that non-alcoholic fatty liver disease (NAFLD) has a strong multifaceted relationship with diabetes and metabolic syndrome, and is associated with increased risk of cardiovascular events, regardless of traditional risk factors, such as hypertension, diabetes, dyslipidemia, and obesity. Given the pandemic-level rise of NAFLD—in parallel with the increasing prevalence of obesity and other components of the metabolic syndrome—and its association with poor cardiovascular outcomes, the question of how to manage NAFLD properly, in order to reduce the burden of associated incident cardiovascular events, is both timely and highly relevant. This review aims to summarize the current knowledge of the association between NAFLD and cardiovascular disease, and also to discuss possible clinical strategies for cardiovascular risk assessment, as well as the spectrum of available therapeutic strategies for the prevention and treatment of NAFLD and its downstream events.

## 1. Introduction

Non-alcoholic fatty liver disease (NAFLD) is the most common chronic liver disease in developed countries. The natural course of the disease moves towards non-alcoholic steatohepatitis (NASH) and cirrhosis, implying that NAFLD will be the leading cause of liver transplantation by 2030 [1]. Beyond the well-known liver-related morbidity and mortality, a large body of evidence has recently shown that patients with NAFLD are also at high risk of cardiovascular (CV) disease, which represents the main cause of death in these subjects [2]. Greater severity of liver disease has been associated with an increased risk of both fatal and non-fatal CV events. Epidemiological and clinical studies have confirmed the role of NAFLD in the development of different CV manifestations, such as left ventricular dysfunction, atherosclerotic CV disease, cardiac conduction system abnormalities and ischemic stroke, suggesting that its contribution may be independent of the presence of traditional CV risk factors [3,4,5]. Despite this evidence, NAFLD is still considered as a benign condition, and clinical practice remains largely unchanged. This review aims to provide a synthesis and critical evaluation of the literature relevant to the postulated continuum between NAFLD and CV disease.

## 2. Epidemiology of NAFLD: The Role of Diet and Lifestyle as Common Risk Factors for CV Diseases

NAFLD affects about 25%–30% of the adult population in Western countries, but its prevalence is increasing also in developing economies, in particular South America and East Asia [6]. The diffusion of a sedentary lifestyle and poor eating habits are the main causes of this change in prevalence, as well as the concomitant growth of metabolic diseases such as obesity and diabetes. In fact, NAFLD prevalence ranges from 50%–75% in subjects with type 2 diabetes [7,8], and, according to different studies, from 80%–90% in obese subjects [9,10].

A low quality diet and high consumption of foods containing saturated fat and processed meat are shared risk factors for the development of NAFLD, metabolic disorders [11,12,13,14], as well as for cardiovascular and all-causes mortality [15,16], confirming the complex interconnection between diet, metabolism, metabolic liver diseases, and the cardiovascular system. By contrast, a correct lifestyle and healthy diet based on both increased physical activity and a higher consumption of fruits and vegetables are associated with a reduced risk of NAFLD development. In post-menopausal women, there is a higher prevalence of NAFLD compared with men, and this difference seems to be mainly related to a higher incidence of metabolic complications and negative cardiovascular profile [17,18]. It has been demonstrated that a lifestyle-induced weight loss is effective to improve not only the histological activity of liver disease in NAFLD patients, but also the cardio-metabolic risk profile [19], defining a possible role of the adequate NAFLD management in reducing CV complications. It is also relevant to note that lifestyle modifications have shown positive effects in NAFLD patients without associated metabolic comorbidities such as obesity [20].

## 3. NAFLD and the Risk of Cardiovascular Events: The Burden of Evidence

The possible role of NAFLD as an adjunctive risk factor for the development of CV diseases has been debated for a long time, and only recent evidence has demonstrated an existing relationship between these conditions. It is well established that NAFLD is associated with an increased prevalence of traditional CV risk factors, in particular type 2 diabetes and obesity [21], but some studies have aimed to evaluate long-term outcomes in patients with NAFLD and observed that the presence of liver disease represents a predictor of CV events, regardless of the association with traditional risk factors. A recent meta-analysis of observational and retrospective studies involving 34,043 adult individuals has shown that the outcome of NAFLD patients is largely affected by cardiovascular events exceeding liver complications, and patients with NAFLD have an increased risk of both fatal and non-fatal CV events compared with patients without NAFLD (odds ratio (OR) of 1.64, 95% CI 1.26–2.13). In this study, a further increase in the risk of CV events (OR 2.58; 1.78–3.75) was observed among patients with a greater severity of liver disease [22]. Interestingly, another finding was observed in the studies (in which the NAFLD diagnosis was obtained by biopsy), namely that there was a significant correlation between the presence of more severe histological forms and a higher risk of CV mortality. Patients with liver fibrosis stage 3–4 demonstrated increased mortality regardless of the NAFLD activity score (HR 3.3, CI 2.27–4.76, *p* < 0.001) [23].

### 3.1. Coronary Heart Disease

It has been observed that NAFLD patients have an increased risk of coronary artery disease compared to the general population (relative risk (RR): 2.26; 95% CI: 1.04–4.92, *p* < 0.001), and, consequently, an increased risk of mortality from CV disease (RR 1.46, 95% CI 1.31–1.64, *p* < 0.001) [24]. As mentioned above, NAFLD seems to impact CV risk regardless of the presence of CV risk factors such as hypertension and dyslipidemia. A Framingham Heart study of 3529 patients undergoing computed tomography (CT) showed that NAFLD was associated with the presence of subclinical markers of atherosclerosis, such as calcium deposits in coronary arteries, even after risk adjustment for other metabolic risk factors [25]. Furthermore, a prospective cohort study showed that patients with a bright liver on ultrasound examination who underwent coronary angiography had a striking prevalence of coronary artery stenosis, again after adjustment for demographic and metabolic factors (adjusted OR 2.31; 95% CI 1.46 to 3.64) [26]. These data were confirmed more recently in a cohort of patients with indication for coronary angiography, where there was a high prevalence of NAFLD (58.2%), and a higher probability of significant coronary stenosis in NAFLD vs. non-NAFLD patients (84.6% vs. 64.1%; *p* < 0.001), which resulted in higher rate of percutaneous coronary intervention among NAFLD patients (68.3% vs. 43.4%; *p* < 0.001) [27]. The finding, of an independent higher prevalence of coronary artery stenosis, is relevant since it seems to influence per se the CV risk in NAFLD patients, as confirmed by the observational study of Targher et al. who enrolled 2103 diabetic patients during a mean follow-up of six and a half years. They observed 219 cases of non-fatal coronary heart disease (myocardial infarction and revascularization procedures), and 121 cases of cardiovascular deaths, with a higher frequency in NAFLD patients, and this association was not affected by adjustment for multiple risk factors (Hazard Ratio, HR 1.96 (1.4–2.7), *p*< 0.001). The authors concluded that NAFLD might give an additional CV risk above what would be expected considering other metabolic risk factors alone [28].

The hypothesis is that more severe liver disease may be responsible for systemic abnormalities, such as persistent inflammation, and for the development of subclinical atherosclerosis. However, a relevant limitation of available studies is the heterogeneity of techniques used for the assessment and staging of NAFLD, including ultrasound, computer tomography and liver biopsy [29].

### 3.2. Structural Cardiac Abnormalities 

NAFLD has been also associated with structural myocardial and valvular abnormalities. In one study, NAFLD patients showed higher prevalence of left ventricular hypertrophy (LVH) compared with those without NAFLD (82% vs. 18%; *p* = 0.01) and this association remained significant after multiple-risk factor adjustment [30]. Furthermore, a higher (three-fold) prevalence of left ventricular diastolic dysfunction (LVD) has been observed, demonstrating that other functional cardiac parameters can be affected [31]. Data from the CARDIA (Coronary Artery Risk Development in Young Adults) study, in which computed tomography was used to assess the hepatic fat infiltration in a community of 2713 participants, confirmed reduced early diastolic relaxation (e’) velocity (10.8 ± 2.6 vs. 11.9 ± 2.8 cm/s, *p* < 0.0001) and higher LV filling pressure (E/e’ ratio: 7.7  ±  2.6 vs. 7.0  ±  2.3, *p* <  0.0001) in NAFLD patients, compared with those without NAFLD [32]. However, it has been demonstrated that yet other heart diseases can be associated: data from the cross-sectional Study of Health in Pomerania (SHIP) found a higher prevalence of aortic valve sclerosis in patients with vs. without NAFLD (36.8% vs. 28.4%; *p* < 0.001) [33], additionally, association between NAFLD and aortic sclerosis was also observed in diabetic patients (adjusted-OR 3.04, 95% CI 1.3–7.3, *p*  =  0.01) [34].

Taken together, these data not only confirm the additive impact of NAFLD on CV outcomes, but also support a possible role of NAFLD as a risk factor for the development of structural heart diseases such valve dysfunction, myocardial hypertrophy and, consequently, development of conditions such as heart failure. An Italian group of investigators found an increased adjusted-risk of mortality in elderly patients with NAFLD that were hospitalized for acute heart failure (adjusted-HR 1.82, 95% confidence intervals 1.22–2.81, *p* < 0.005) [35]. The NAFLD fibrosis score has been demonstrated to be correlated with a worse outcome in 492 patients with heart failure and preserved ejection fraction [36].

### 3.3. Arrhythmias

Several studies have also suggested a possible role of NAFLD in the development of cardiac rhythm disturbances, and a recent meta-analysis involving two cross-sectional and three cohort studies including a total of 238,129 subjects found a higher risk of atrial fibrillation (AF) in NAFLD patients than in those without liver disease (pooled RR of 2.06, 95% confidence interval-CI-, 1.10–3.85), although a significant heterogeneity was found among the different studies [37]. These data were confirmed in the large SHIP study, where an increase in AF prevalence in patients with elevated serum liver enzymes was found, usually considered as surrogate biomarkers of steatohepatitis (adjusted ORs of 1.65, (95% CI: 1.16 to 2.35; *p* = 0.006) for ALT, 1.47 (95% CI: 1.07 to 2.02; *p* = 0.017) for AST, and 2.17 (95%CI: 1.64 to 2.87; *p* < 0.001) for GGT) [38]. The significant correlation with AF persisted after adjustment for other risk factors (adjusted OR, 1.88, 95% CI 1.03–3.45) in the Oulu Project Elucidating Risk of Atherosclerosis (OPERA) study [39]. This independent association was again confirmed in 400 diabetic patients with ultrasound-documented liver disease, who had a higher risk of incident AF (OR 4.49, 95% CI 1.6–12.9, *p* < 0.005). This relationship persisted after adjustment for other ECG abnormalities such as left-ventricular hypertrophy and P-R interval, age and hypertension (adjusted OR of 6.38, 95% CI, *p* = 0.005) [40]. It is possible that the occurrence of AF is related to the above mentioned left ventricular filling abnormalities. Moreover, NAFLD patients show an increased prevalence of other cardiac rhythm disturbances such as premature ventricular beats (19.3% vs. 6.5% in patients without NAFLD, *p* < 0.005) and non-sustained ventricular tachycardia (14.7% vs. 4.3%, *p* < 0.005). The increased risk of ventricular tachycardia remained significant after adjustment for other risk factors (adjusted OR 3.01, 95% CI 1.26–7.17; *p* = 0.013) [41]. More recently, a retrospective study involving diabetic patients with NAFLD found an increased risk of heart block in patients with NAFLD, compared with those without NAFLD (adjusted OR 3.04, 95% CI 1.81–5.10) [42].

### 3.4. Cerebrovascular Events

An increased risk of cerebrovascular disease has been found in patients with NAFLD. A meta-analysis found an increased risk of ischemic (OR = 2.51, 95% CI 1.92–3.28, *p* < 0.001) and hemorrhagic stroke (OR = 1.85, 95% CI 1.05–3.27, *p* = 0.034) among patients with NAFLD [43], and greater severity of liver disease increased the risk. The degree of liver fibrosis, assessed as liver stiffness on transient elastography, was independently associated with the risk of ischemic stroke (OR ranging between 3.655 and 4.577 for mild fibrosis and from 6.160 to 13.184 for significant fibrosis; all *p* < 0.05) [44].

Furthermore, the presence of NAFLD seems to affect the clinical status of patients who had a cerebrovascular event, confirming its negative role in the outcome of affected subjects. Abdeldyem et al. prospectively evaluated 200 patients with ischemic stroke, and they found that NAFLD was associated with a more negative National Institutes of Health Stroke Scale (NIHSS) score at the hospital admission, and a higher disability on discharge, as evaluated with the Modified Rankin Scale score (*p* < 0.05 for each comparison) [45]. These results confirm the close relationship between NAFLD and the cerebrovascular system [46].

### 3.5. Thromoboembolic Events

Liver diseases are well known to be responsible for disturbances of coagulation. In particular, severe hepatic damage is associated with reduced production of coagulation factors, resulting in an increase of hemorrhagic manifestations. Less frequently, cirrhotic patients can develop portal vein thrombosis. NAFLD patients can show a persistent, systemic prothrombotic state, which may account for a higher risk of thrombotic -more than hemorrhagic- complications [47]. A recent study showed that non-alcoholic steatohepatitis (NASH)-related cirrhosis is associated with an increased risk for thromboembolism, even after adjustment for confounding factors (OR: 2.46, 95% CI: 1.07–5.65, *p* = 0.034) [48].

## 4. Cardiovascular Risk by Subtypes of NAFLD

The definition of NAFLD encompasses a spectrum of different clinical-pathological entities with different degrees of severity characterized by the tendency to evolve from less severe to more severe forms: steatosis or non-alcoholic fatty liver (NAFL), NASH, advanced fibrosis-cirrhosis and, finally, hepatocellular carcinoma (HCC) [49]. As mentioned before, an increasing body of evidence supports the hypothesis of different CV risks among the different classes of severity of NAFLD. Liver inflammation may represent the main determinant of systemic complications in NAFLD subjects; therefore, its evaluation could be useful to assess the CV risk. In patients with NAFLD, the presence of overt steatohepatitis at biopsy was associated with cardiac abnormalities such as left atrium enlargement and increased left ventricular mass compared with those without fatty liver disease [50]. Further, a correlation between the severity of hepatic steatosis on ultrasound and the CV risk, measured with the Framingham Risk Score, has also been demonstrated [51]. Given the reported association between ultrasonographic severity of steatosis and coronary/carotid atherosclerotic disease, liver ultrasound may assist in identifying subjects at high CV risk [52]. Fibrosis is a complication observed in the advanced stages of liver disease, and it is a well-known risk factor for increased mortality. This data has been confirmed in patients with biopsy-proven NAFLD, in which the increased risk of mortality was correlated with a fibrosis stage of 3–4 (HR of 3.3, CI 2.27–4.76, *p* < 0.001) [23].

The evidence of increase in CV risk in patients with NAFLD suggests adoption of early intervention with the aim of reducing the risk of disease progression and improving the cardiovascular outcome.

## 5. Pathophysiology of NAFLD and Associated Cardiovascular Abnormalities

The mechanisms by which NAFLD increases CV risk are very complex and involve simultaneously different pathways at different functional and structural levels, namely the metabolism, cardiovascular system, and liver function. Insulin resistance is recognized as the main determinant of NAFLD pathogenesis but as to how NAFLD is responsible for accelerated atherosclerosis, independently of metabolic abnormalities, remains unclear. Probably, the hepatic necro-inflammation with the consequent systemic diffusion of cytokines and chemokines leads to vascular damage and coagulation system abnormalities. It is also possible that NAFLD and CV diseases share a common inherited predisposition, which further influences the CV risk in these patients.

## 6. Genetics

Genetic factors seem to play a key role in both the development and severity of NAFLD, with a percentage of heritability of about 27% in the Caucasian population [53]. Similar results have been observed also among Hispanics and African Americans, despite the presence of some differences between the various populations (33% Hispanic and 14% African Americans) [54,55]. Polymorphisms of patatin-like phospholipase domain containing-3 (*PNPL3*) gene have been recognized as the most relevant genetic risk factor for NAFLD [56,57]. Interestingly, some evidence has suggested a possible role of this genetic variant also in the susceptibility to CV disease. In particular, an Italian study has shown that, in young patients with a histological diagnosis of NAFLD, the presence of PNPLA3 GG polymorphism was associated with a higher prevalence of carotid plaques and intima-media thickening compared with the CC/CG genotype (53% vs. 32%, *p*  =  0.02; 62%vs.28%, *p* < 0.001, respectively), but also with intima-media thickening progression during follow-up [58]. PNPLA3 is a transmembrane hydrolase that is highly expressed in adipocytes and hepatocytes and is involved in the metabolism of triglycerides, therefore its genetic variation may promote the development and progression of atherosclerotic plaques. In addition, other PNPLA3 polymorphisms have been associated with increased inflammatory activity of the endothelium, in particular the rs738409 polymorphism seems to be associated with abnormal circulating levels of ICAM-1, an endothelium-derived inflammatory molecule [59]. Other genes have been associated with the development of NAFLD, such as the polymorphism of the transmembrane 6 superfamily member 2 (TM6SF2), a gene encoding a protein whose global function is still unknown but is probably involved in the hepatic lipid metabolism. However, some variants of this gene seem to be associated with different effects (protective vs. negative) on the CV system [60,61]. Other genetic polymorphisms have preferential deleterious effects, such as the variant encoding for Glu167Lys which has been associated with a higher risk of myocardial infarction [62]. Finally, some genetic polymorphisms are associated with a high susceptibility to CV disease in patients with NAFLD. Variants of genes encoding for adiponectin or leptin receptors have been associated with more severe liver damage but also with severe dyslipidemia, diabetes and obesity, confirming a predisposition to shared cardio-metabolic complications in NAFLD [63,64,65,66].

### 6.1. Adipose Tissue and Dyslipidemia: Linking Points between NAFLD and CV Disease

Considering the close relationship between metabolic diseases, such as metabolic syndrome and obesity, and the development of NAFLD, the adipose tissue expansion and dysfunction is probably the most significant common point between liver disease and cardio-metabolic complications. In fact, overeating and excessive calorie intake are usually the main determinants of metabolic syndrome, NAFLD pathogenesis, and CV manifestations, leading to increased serum levels of free fatty acids (FFA) that exceed the storing-capacity of adipose tissue with consequent fat mass enlargement, visceral and ectopic fat deposition, also involving the liver. The abnormal expansion of adipose tissue leads in turn to adipocyte dysfunction with persistent production of adipose-derived cytokines (interleukin-6, tumor necrosis factor α) and C-reactive protein. The presence of persistent inflammation can be associated not only with the development of insulin resistance, but also with the direct onset of CV diseases [67].

Furthermore, increased lipogenesis, due to enlarged fat mass, is responsible for high circulating levels of FFA, VLDL overproduction, and further lipid metabolism abnormalities, which can lead to significant atherogenic dyslipidemia. The dyslipidemia in NAFLD patients may have a more severe atherogenic potential, with higher serum levels of small dense LDL, lower HDL, and a higher prevalence of hypertriglyceridemia. Moreover, a positive correlation between lipid parameters and histological severity of liver damage and inflammation has been demonstrated in NAFLD patients [68], confirming the strong relation between the two conditions.

### 6.2. Microvascular Damage, Endothelial Dysfunction and CV Risk in NAFLD Patients

The presence of systemic microvascular damage, along with impaired endothelial dysfunction due to persistent inflammation, and oxidative stress could be some of the main mechanisms which increase CV risk in the NAFLD population [69]. Beyond the direct effect of increased lipogenesis, increased arterial stiffness can promote the CV risk in NAFLD patients [70]. NAFLD patients show increased prevalence of arterial rigidity and consequent hypertension, and the degree of arterial dysfunction is higher in patients with NASH [71]. The evidence of increased arterial stiffness in NAFLD patients has been demonstrated indirectly by the finding of a positive correlation between the hepatic artery resistive index, an ultrasound parameter which assesses the rigidity of hepatic artery and the NAFLD fibrosis score, a non-invasive scoring system which is useful to predict the amount of scarring in the liver [72,73].

Furthermore, the evidence of a higher prevalence of NAFLD in non-dipper patients, and in general with those with altered dipping status [74], along with the evidence that the severity of NAFLD seems to increase simultaneously with the incidence of arterial hypertension in this population, support the strong, dual relation that there is between the two conditions [75].

### 6.3. Liver-Specific Abnormalities and Cardiovascular System

As mentioned above, beyond the direct influence of comorbidities such as atherogenic dyslipidemia, obesity and arterial hypertension on the CV system, liver disease seems to significantly and directly promote CV risk. The presence of impaired intrahepatic vascular function (sinusoid distortion, loss of fenestration or microvascular formation) and fibrosis can lead to endothelial dysfunction per se and to increased production of pro-thrombotic molecules and angiogenic factors which may involve the systemic vasculature [76]. The liver contains the largest number of residential macrophages, and a high number of cytokines (in particular TNF-α, IL-6, CRP) can be chronically released into the systemic circulation, promoting chronic inflammation and thrombotic susceptibility [77].

NAFLD patients may have important liver vascular remodeling that could contribute to arterial dysfunction and CV risk. Increased serum levels of vascular-endothelial growth factor (VEGF) have been found in NAFLD patients compared with controls [78]. A high production of pro-thrombotic factors, in particular factors VIII, IX, XI and XII, has been positively correlated with hepatic fat content, and therefore can be associated with an increase in CV risk in these patients [79]. Increased release of the plasminogen activator inhibitor-1 (PAI-1), which is associated with prothrombotic risk by inhibiting the tissue plasminogen activator, can further contribute to the risk of cardiovascular events [80].

Recently, it has also been demonstrated that there is a systemic role for hepatic tissue-specific molecules, which seem to affect multiple metabolic pathways. These molecules, also called “hepatokines”, may have a relevant role in the development of cardiovascular complications in patients with NAFLD. The fibroblast growth factor-21 (FGF-21) is a peptide secreted by the liver and is involved in human homeostasis. Its role in is not completely understood, but some studies have shown negative effects on the cardiovascular system. Increased serum levels of FGF-21 have been associated with carotid intima-media thickening, atherosclerosis [81], and coronary artery disease [82]. Fetuin-A is a molecule synthesized by the liver which appears to be involved in insulin signaling. A negative effect on the CV system has been observed in diabetic patients with higher levels of fetuin-A, while opposite effects were observed in non-diabetic patients [83].

An altered status of human gut microbes has been described both in NAFLD and CV disorders, such as myocardial infarction and stroke [84,85,86,87]. Experimental studies have shown that changes in gut microbes may affect natural homeostasis, in particular, by reducing energy expenditure and insulin sensitivity. In humans, abnormal intake of food has been associated with the development of obesity, diabetes and NAFLD, with influence on the gut microbial composition. The trimethylamine N-oxide, a molecule which is converted from dietary phosphatidylcholine by gut microbes, has been associated with the development of atherosclerotic disease [88,89]. In addition, a significant amount of enterobacteria DNA, such as Proteobacteria, has been demonstrated in atherosclerotic plaque [90]. These findings suggest the role of intestinal dysbiosis at the crossroad of diet, metabolic disease, NAFLD and cardiovascular events.

**Figure 1:**
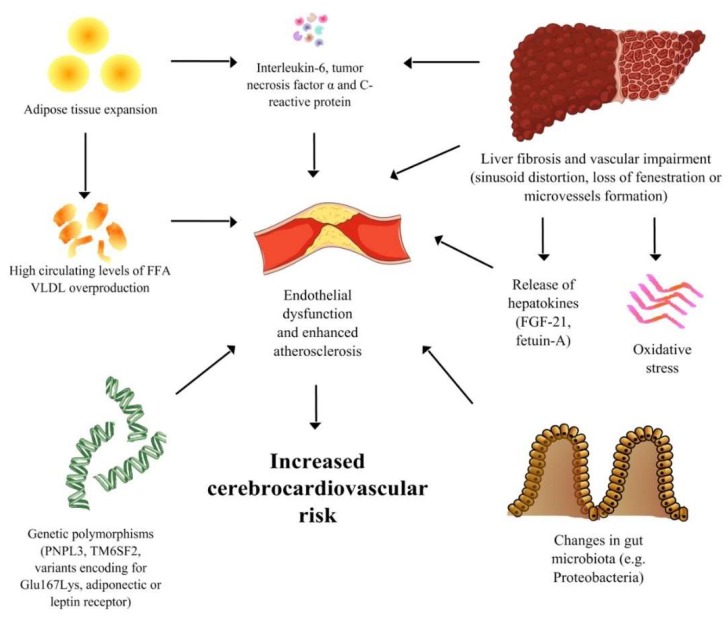
A Spotlight on the Pathogenesis of Cardiovascular Risk Associated with Non-Alcoholic Fatty Liver Disease.

Figure 1 shows the multiple mechanisms of connection between NAFLD and CV disease.

## 7. Specific Cardio-Metabolic Risk Assessment and Follow-Up of NAFLD Patients

### Cardiovascular Risk Assessment in NAFLD Patients

Considering the effects of NAFLD on the CV system, it could be useful to assess the cardio-metabolic profile of NAFLD patients in order to identify people at higher risk of CV disease and CV mortality. Careful assessment of CV family history should be obtained, and a thorough clinical evaluation of the patient, with an assessment of physical status and laboratory parameters such as blood pressure, waist circumference, BMI, blood cholesterol and triglycerides levels, glomerular filtration rate, and albuminuria, is important to recognize early the presence of metabolic alterations. Considering the strong association between NAFLD and diabetes [91], random blood glucose and/or glycosylated hemoglobin should also be measured in patients without a history of diabetes. Some cardiovascular risk assessment tools, in particular the American College of Cardiology/American Heart Association tool and the Framingham risk score, have been used in patients with NAFLD and are valuable to identify those at higher risk of cardiovascular events at 10 years [92,93]. Beyond the identification of NAFLD, imaging assessment of sub-clinical markers of CVD, such as the presence of carotid intima-media thickening, coronary calcifications, and left ventricular dysfunction, could be useful to modulate the intensity of treatment. In this regard, the correct identification of liver damage (NAFL vs. NASH vs. overt cirrhosis) should be combined for ideal risk stratification. Considering the burden of CV and metabolic complications, we suggest this approach to identify patients at high risk and to intensify preventive measures where appropriate.

## 8. Treatment of NAFLD: Measures Aimed at Reducing both Liver Disease and CV Risk

Presently, there is no licensed pharmacological treatment for NAFLD [94].

A tailored multistep treatment approach has been proposed for the management of NAFLD as a tool effectively to reduce the risk of CV disease in these patients by treating the co-existing features of the metabolic syndrome (MetS) [94]. The first fundamental step is attaining favorable lifestyle changes by means of an appropriate diet, physical exercise and smoking cessation. Further steps that may lead to bariatric surgery should, however, be primarily reserved for patients with NASH/NAFLD-associated high-risk metabolic comorbidities such as obesity, dyslipidemia, hypertension and type 2 diabetes (T2D).

### 8.1. Lifestyle Changes

A combination of diet and physical exercise is universally recommended as the first-line approach to the management of NAFLD [94].

A large prospective study has shown that weight loss, induced by diet alone or combined with physical exercise, is associated with significant improvement in histological features of NAFLD in proportion to the amount of body weight reduction (at least 3%–5% weight loss reduces hepatic steatosis while a weight loss greater than 7–10% is needed to improve hepatic necro-inflammatory activity and fibrosis) [95]. Unfortunately, the percentage of patients who achieved the 7–10% weight loss goals was very low. Interestingly, exercise alone exerts beneficial effects on serum liver enzymes and extent of steatosis even in the absence of weight loss, but its ability to improve other aspects of liver histology remains unknown [96].

Diet and physical exercise can lead to real CVD risk reduction by improving atherogenic risk profile and myocardial structure and function. Moreover, the latter may exert beneficial cardiovascular effects by inducing anti-inflammatory activity [5].

The optimum macronutrient diet composition for NAFLD is not known [97]. A low-calorie (about 1200–1600 kcal/day) low-fat low-carbohydrate diet has been recommended, notably by reducing saturated fats and simple carbohydrates [98]. Soft drinks should be avoided in NAFLD patients given the strong association between high fructose consumption and metabolic syndrome, the development and progression of NAFLD [98]. A recent, large, and randomized controlled trial reported significant reduction in the incidence of CVD events in subjects consuming a “Mediterranean” diet supplemented with extra-virgin olive oil or nuts compared with those following a low-fat diet [99]. Results from the available studies suggest that the Mediterranean diet is effective in patients with NAFLD but there is still need for trials with larger sample sizes [100,101].

Light-to-moderate alcohol consumption may provide potential cardiovascular benefits in the general population, but there is insufficient evidence for or against light-to-moderate alcohol consumption in NAFLD [102].

NAFLD patients should be strongly advised to stop smoking, an established CVD risk factor, which has also been associated with development and progression of NAFLD [103].

### 8.2. Antidiabetic Drugs

#### Insulin Sensitizers

Metformin, the first-line oral treatment of type 2 diabetes, has been proven to reduce the risk of HCC moderately but does not improve liver histology in patients with NAFLD [104].

Pioglitazone, a peroxisome proliferator-activated receptor (PPAR)-gamma agonist, ameliorates systemic insulin resistance and improves histological features of NAFLD/NASH, namely steatosis, necro-inflammatory activity and also advanced fibrosis according to recent evidence [105]. Unfortunately, hepato-protective effect of glitazones has been reported to be short-lived after drug discontinuation. Pioglitazone modestly reduces major CVD events in subjects with T2DM but causes significant weight gain (by increased subcutaneous adipose tissue), and increases the risk of congestive heart failure, bone fractures and slightly of bladder cancer [106,107]. Therefore, the potential hepatic benefits of pioglitazone use should be weighed against its lack of benefits for the cardiovascular system, suggesting that the reduction of CVD risk should be based on a more global approach than just glucose control [108]. In conclusion, pioglitazone use should be limited to patients with advanced NASH and T2DM, without heart failure or other contraindications to glitazones.

### 8.3. Incretin Based-Therapy

#### Glucagon-like Peptide-1 Receptor Agonists

Glucagon-like peptide-1 receptor agonists (GLP-1RAs) (exenatide, lixisenatide, liraglutide and semaglutide) due to their hypoglycemic effects reduce appetite promoting body weight loss [109].

A recent small multicenter study on subjects with biopsy-proven NASH has shown that liraglutide was safe and led to resolution of NASH (defined as disappearance of hepatocyte ballooning) in 39% of patients (versus 9% in placebo group, *p* = 0.019), while no significant differences were observed in progression of fibrosis between two groups. Drug-associated weight loss, together with a direct hepatic effect, may have mediated the beneficial histological changes [110].

### 8.4. Dipeptidyl Peptidase-4 Inhibitors

Few studies are available on the effect of DPP-4 inhibitors (DPP-4Is) such as sitagliptin and vildagliptin in NAFLD patients [109]. A recent randomized controlled trial showed that sitagliptin was ineffective in reducing liver fat, assessed by magnetic resonance imaging (MRI), and liver enzymes compared with placebo [111].

### 8.5. Sodium-Glucose Transporter 2 Inhibitors

Sodium-glucose transporter 2 inhibitors (SGLT2Is) (empaglifozin, dapaglifozin, remoglifozin etabonate) exert antidiabetic action by blocking renal glucose reabsorption resulting in markedly increased glycosuria. Recent findings from small randomized controlled trials suggest that treatment with dapaglifozin vs. placebo in patients with NAFLD and T2DM is associated with improvement in liver fat content (assessed either by controlled attenuation parameter or MRI), hepatocyte injury biomarkers and attenuation of significant fibrosis (assessed by liver stiffness) [112,113].

Available data from cardiovascular outcomes trials suggest that long-acting GLP-1RAs (liraglutide and semaglutide) reduce major adverse cardiovascular events in subjects with T2DM at high risk. In contrast DPP-4Is seem to have a neutral effect on cardiovascular outcomes and some concerns have been raised about increased risk of hospitalization due to congestive heart failure with saxagliptin (significant) and alogliptin (non-significant trend), suggesting caution in using DPP-4Is in individuals at high risk of heart failure [114]. Conversely, empagliflozin has shown to reduce cardiovascular mortality and hospitalization for congestive heart failure in similar populations of patients with T2D at high risk for cardiovascular events [115].

In conclusion, limited data suggest a potential benefit of GLP-1RAs and SGLT2Is on both liver and cardiovascular system. However, further larger and longer randomized clinical trials with histological endpoints are needed to establish a beneficial effect of these drugs for the treatment of NAFLD/NASH.

### 8.6. Lipid Lowering Agents

#### Statins

Statins, inhibitors of the cholesterol synthesis, are the most widely used and effective lipid lowering drugs for the primary and secondary prevention of CVD and their use has proven to be safe in NAFLD patients [116,117]. Statins may improve liver enzymes, steatosis and necroinflammation but not fibrosis in NAFLD patients [118,119]. Moreover, epidemiological studies suggest that statins are associated with reduced risk of HCC [120].

Ezetimibe, another lipid-lowering agent which reduces the intestinal uptake of dietary cholesterol, may exert some improvement in NAFLD histology [121]. The benefits of the combination of statins and ezetimibe on CVD risk profile have been widely reported in the literature [122].

The specific beneficial cardiovascular effect of statins in NAFLD patients is supported by two studies showing that the relative reduction of CVD risk was higher in patients with mildly-to-moderately abnormal liver aminotransferases, potentially attributable to NAFLD [123,124]. Consistently, a recent sub-analysis of the IMPROVE-IT trial has shown a greater benefit of combination therapy with simvastatin plus ezetimibe in high CV risk patients defined by high NAFLD-fibrosis score (NFS > 0.676) [124].

These findings suggest that moderately abnormal transaminases as well as high NFS may be useful markers for identifying subjects at particularly high CVD risk and thus specifically needing aggressive pharmacological intervention.

### 8.7. Fibrates

Fibrates lower serum triglyceride levels while simultaneously increasing HDL-cholesterol levels, by activating PPAR-alpha [122]. Fibrates reduce CVD morbidity and mortality only in patients with atherogenic dyslipidemia [122]. Although fibrates have not been shown to improve NAFLD histology, they do offer a safe and effective treatment for atherogenic dyslipidemia in NAFLD patients, especially those with MetS and/or T2DM [4].

### 8.8. Omega-3 Polyunsaturated Fatty Acids

Recent studies have failed to show convincing histological improvement in NAFLD/NASH in subjects treated with omega-3 polyunsaturated fatty acids (PUFAs) [97,125] Omega-3 PUFAs may be considered to treat hypertriglyceridemia in NAFLD patients [97]. However, a recent Cochrane metanalysis has shown that omega 3 PUFAs have little or no benefit in both primary and secondary prevention of CVD [126].

### 8.9. Angiotensin Receptor Blockers

The renin-angiotensin-aldosterone system is involved in the pathogenesis of insulin resistance, NAFLD and target organ damage [5].

Some experimental studies reported that angiotensin receptor blockers (ARBs) might improve serum liver enzyme levels and histologic features of NAFLD. However, current evidence is insufficient to support the efficacy of ARBs in managing fibrosis in NAFLD patients [127,128]. It is well established that ARBs reduce blood pressure values and also improve glucose control, thus leading to further reduction of CVD events by preventing new-onset T2DM [129].

### 8.10. Vitamin E

Given the shared role of oxidative stress in the pathogenesis of both NASH and atherosclerosis, antioxidants and specific vitamins may in theory improve liver histology and reduce CVD risk. Vitamin E administered at a daily dose of 800 IU/day improves liver histology in nondiabetic adults with biopsy-proven NASH [130] and therefore may be considered for treating these patients. However, concerns have been raised on the long-term safety of vitamin E [131] and the lack of data in patients with T2DM and cirrhosis, thus, is not recommended in these groups. Moreover, current evidence suggests that vitamin E supplementation has no role for the treatment/prevention of CV disease [132].

### 8.11. Bariatric Surgery

Bariatric surgery, which should be reserved for morbidly obese patients who are non-responders to lifestyle interventions, has been associated with beneficial and sustained improvements in liver histology in people with NAFLD/NASH [133]. Moreover, it provides strong cardiovascular benefits, reducing CVD and overall long-term mortality through the prevention of diabetes, improvement of cardio-metabolic risk factors, cardiac morphology, and function [134,135].

## 9. Conclusions

Given the increased risk for CVD observed in NAFLD patients, and the strong bidirectional association of NAFLD with metabolic syndrome, we believe that all cardiometabolic risk factors should be carefully and routinely screened among patients with NAFLD, and that disease management should be focused on both specific lifestyle modifications and aggressive risk factors modification, which would not only reduce the risk of liver disease progression, but may also provide benefit by reducing the risk of developing cardiac complications. Strict follow-up should be ensured to monitor the adhesion, tolerability, and impact of treatment interventions in NAFLD patients. Randomized controlled trials with a long-term follow-up are eagerly awaited to ascertain whether effective NAFLD treatment will translate into better CV outcomes.
